# Evidence of star cluster migration and merger in dwarf galaxies

**DOI:** 10.1038/s41586-025-08783-9

**Published:** 2025-04-09

**Authors:** Mélina Poulain, Rory Smith, Pierre-Alain Duc, Francine R. Marleau, Rebecca Habas, Patrick R. Durrell, Jérémy Fensch, Sungsoon Lim, Oliver Müller, Sanjaya Paudel, Rubén Sánchez-Janssen

**Affiliations:** 1 Space Physics and Astronomy Research Unit, University of Oulu, Oulu, Finland; 2 Universidad Técnica Federico Santa María, Santiago, Chile; 3 Université de Strasbourg, CNRS, Observatoire astronomique de Strasbourg, Strasbourg, France; 4 Institut für Astro- und Teilchenphysik, Universität Innsbruck, Innsbruck, Austria; 5 INAF - Astronomical Observatory of Abruzzo, Teramo, Italy; 6 Department of Physics, Astronomy, Geology and Environmental Sciences, Youngstown State University, Youngstown, OH USA; 7 University of Lyon, ENS de Lyon, CNRS, Centre de Recherche Astrophysique de Lyon, Lyon, France; 8 Department of Astronomy, Yonsei University, Seoul, Republic of Korea; 9 Institute of Physics, Laboratory of Astrophysics, École Polytechnique Fédérale de Lausanne (EPFL), Sauverny, Switzerland; 10 Institute of Astronomy, Cambridge, UK; 11 Clare Hall, University of Cambridge, Cambridge, UK; 12 Department of Astronomy and Center for Galaxy Evolution Research, Yonsei University, Seoul, Republic of Korea; 13 UK Astronomy Technology Centre, Royal Observatory Edinburgh, Edinburgh, UK

**Keywords:** Galaxies and clusters, Computational astrophysics

## Abstract

Nuclear star clusters (NSCs) are the densest stellar systems in the Universe. These clusters can be found at the centre of all galaxy types but tend to favour galaxies of intermediate stellar mass around 10^9^*M*_⊙_ (refs. ^1,2^). At present, two main processes are under debate to explain their formation: in situ star formation from gas infall^3^ and migration and merging of globular clusters (GCs) caused by dynamical friction^4^. Studies^5–9^ of NSC stellar populations suggest that the former predominates in massive galaxies, whereas the latter prevails in dwarf galaxies, and both contribute equally at intermediate mass. However, until now, no ongoing merger of GCs has been observed to confirm this scenario. Here we report the serendipitous discovery of five dwarf galaxies with complex nuclear regions, characterized by multiple nuclei and tidal tails, using high-resolution images from the Hubble Space Telescope. These structures have been reproduced in complementary *N*-body simulations, supporting the interpretation that they result from migrating and merging of star clusters. The small detection rate and short simulated timescales (below 100 Myr) of this process may explain why this has not been observed previously. This study highlights the need for large surveys with high resolution to fully map the migration scenario steps.

## Abstract (web summary)

High-resolution images from the Hubble Space Telescope show evidence of star cluster migration and merger in dwarf galaxies.

## Main

Witnessing the formation of nuclear star clusters (NSCs) is important to fully understand how these extremely compact and massive objects can form at the centre of a wide range of galaxy types. NSCs, typically, have a stellar mass M_*_ in the range of 10^5^–10^8^*M*_⊙_ and an effective radius *R*_e_ up to several tens of parsecs (ref. ^10^). Over the past decades, two main scenarios have been put forward and are debated to explain the formation of NSCs.

In the in situ formation scenario^3^, the infall of gas triggers star formation and forms an NSC. This scenario is favoured in massive galaxies and predicts the presence of two compact sources: a massive black hole or a star cluster surrounded by a stellar disk and a compact object formed from stars gathering at the disk apoapsis. This is observed in the nuclear region of the Andromeda galaxy^11^ and has been reported in several other galaxies^12–14^.

Alternatively, NSCs could form from the infall of GCs due to dynamical friction^4^. This migration plus merging scenario is suggested to dominate in dwarf galaxies. Observational signatures of this scenario include the presence of multiple star clusters^15–17^ and tidal interactions in the inner regions, possibly leading to the formation of tails. However, no direct observation of tidally interacting and merging star clusters near the centre of dwarf galaxies has been reported so far in the literature.

The study of the stellar population alone is often not enough to differentiate between the two scenarios. Young stellar populations, in particular, can result from either formation scenario or a combination of the two. An example of this combination is the wet-merger scenario^18^, that is, the formation of an NSC from the migration of a star cluster together with a gas reservoir. Thus, these studies need to be coupled with high-resolution observations to fully reconstruct the formation steps of NSCs.

An NSC growth scenario is possible by involving interacting galaxies. In the stages of a merger between two nucleated galaxies, multiple nuclei should be visible in the central regions of the galaxies, as observed in several systems^19^. The nuclei will eventually end up merging to form the NSC of the galaxy remnant. A clear sign of ongoing or past galactic collisions is the presence of an overall boxy shape of the galaxy remnant, or tidal tails and shell structures in its outskirts.

**Extended Data Fig. 1:**
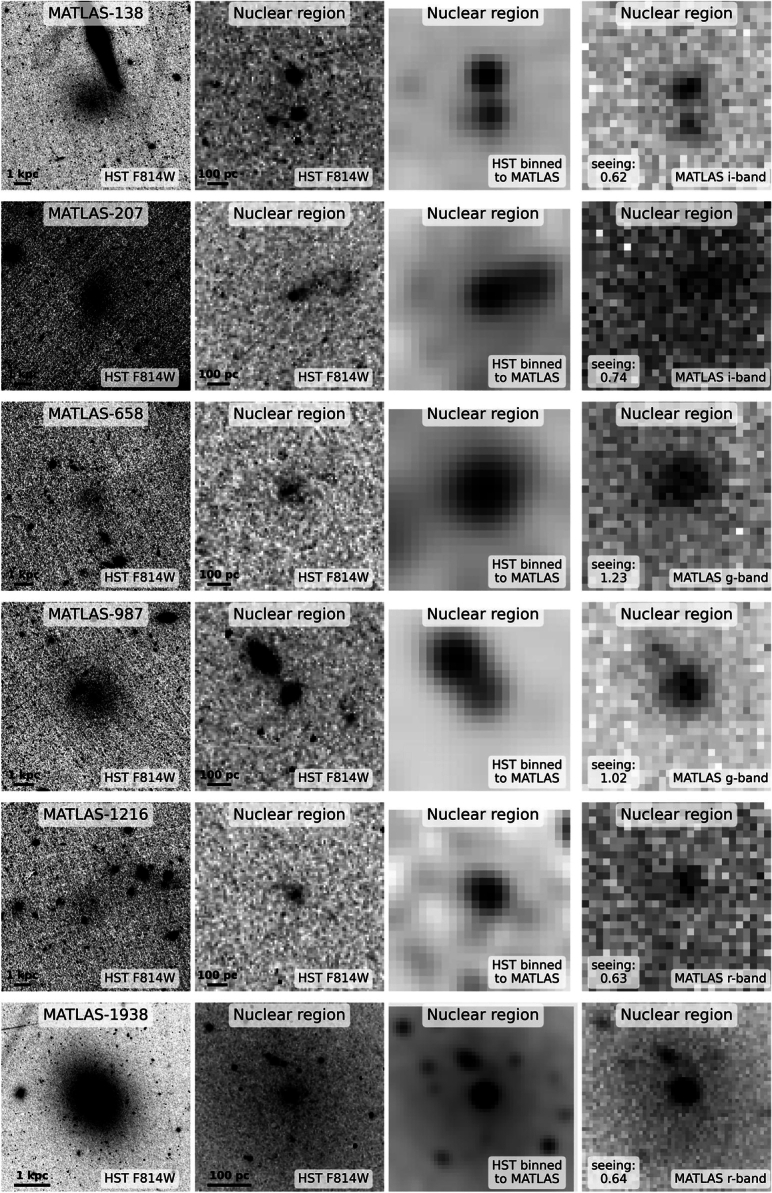
The six non-interacting dwarf galaxies showing a complex nucleus. For each galaxy, *from left to right*: 1′ × 1′ cutout of the dwarf in the HST F814W filter, the central 5″ × 5″ region of the dwarf, the nuclear region cutout 4pix × 4pix binned to which we applied a Gaussian filter to obtain a seeing similar to MATLAS, and the corresponding MATLAS observation in the band with the best seeing. MATLAS-1938 has a 10″ × 10″ zoom into its nuclear region. All images have North up and East left.

**Extended Data Fig. 2:**
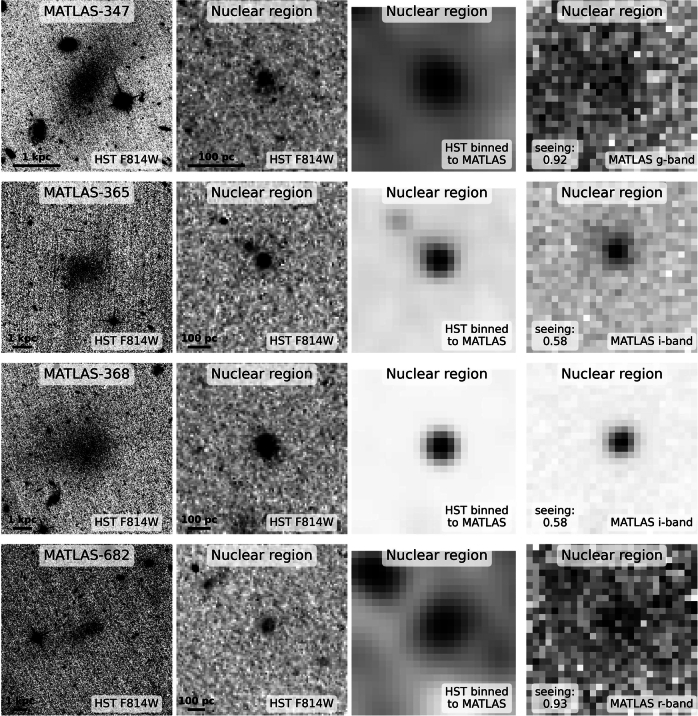
The four dwarf-dwarf merger candidates showing a complex nucleus. For each galaxy, *from left to right*: 1′ × 1′ cutout of the dwarf in the HST F814W filter, the central 5″ × 5″ region of the dwarf, the nuclear region cutout 4pix × 4pix binned to which we applied a Gaussian filter to obtain a seeing similar to MATLAS, and the corresponding MATLAS observation in the band with the best seeing. All images have North up and East left.

High-resolution optical images of a sample of 79 dwarf galaxies were recently obtained with the Hubble Space Telescope (HST) as part of follow-up observations of nearby galaxy satellites from the Mass Assembly of Early-Type Galaxies with Their Fine Structures (MATLAS) survey (Methods). Most of the selected galaxies for the HST follow-up have a lower surface brightness and a larger size than typical dwarfs and can be defined as ultra-diffuse galaxies^20^ (UDGs). The HST sample covers about 65% of the MATLAS UDG sample^21^, and apart from their size and surface brightness criteria, UDGs have similar structural properties compared with dwarfs. Among the galaxies observed with HST, 10 exhibit a nucleus with substructure, such as multiple star clusters and stellar tidal tails. We note that bright sources are observed on the deep ground-based MATLAS images at the location of the complex nuclear systems revealed by the high-resolution HST images (Extended Data Figs. 1 and 2). This excludes the possibility that these nuclear substructures are instrumental artefacts.

**Fig. 1:**
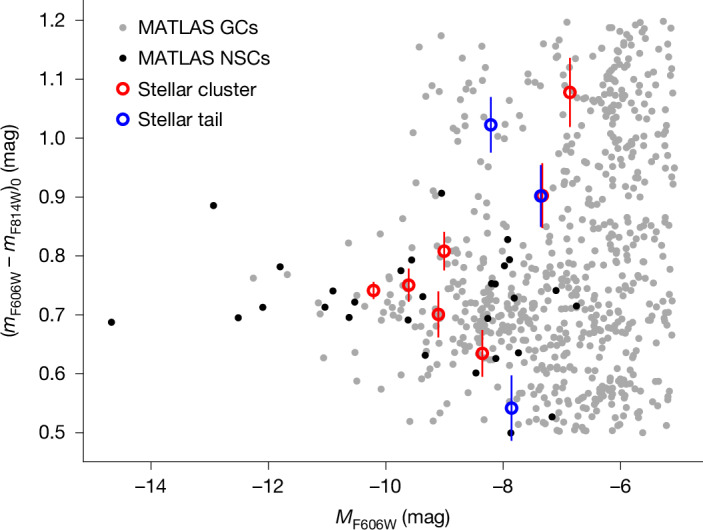
Colour–magnitude diagram of the substructures of the five nuclear regions consistent with a migration scenario origin. We compare the star clusters (red circles) and tails (blue circles) of their complex nuclear regions to GCs (grey dots) and NSCs (black dots) of the MATLAS dwarfs observed by HST. The error bars correspond to the standard error combined with the standard deviation of the sky.

To check whether the observed nuclear substructures are signatures of the migration scenario, that is, the predicted formation process for the nucleus of dwarf galaxies, we isolated a sample of dwarfs whose nuclear structures probably formed from internal mechanisms and whose luminosity and colour are consistent with NSCs and GCs from the MATLAS survey (Methods). Therefore, we removed four dwarf–dwarf merger candidates (Extended Data Fig. 2) and excluded one NSC that might be experiencing a wet-merger scenario given the blue colour of some structures of its nucleus (Extended Data Table 1). Hence, we are left with a sample of five galaxies. A colour–magnitude diagram of their substructures is shown in Fig. 1.

We computed the detection rate of interacting–merging nuclei in our specific sample of dwarfs observed with the HST. Of the whole initial sample, only 13% (10/79) show a complex nuclear region. Moreover, the fraction decreases to 7% (5/74) when we exclude the dwarf–dwarf merger candidates and to 4% (3/74) if we focus only on the least ambiguous sign of a star cluster merger—tidal tails. This detection rate might be higher than that of typical dwarfs because the low central surface brightness of our galaxies makes the detection of tidal tails easier. Thus, observing complex nuclei, and especially tidal tails, is rather rare and requires a large sample of low surface brightness dwarfs.

**Fig. 2:**
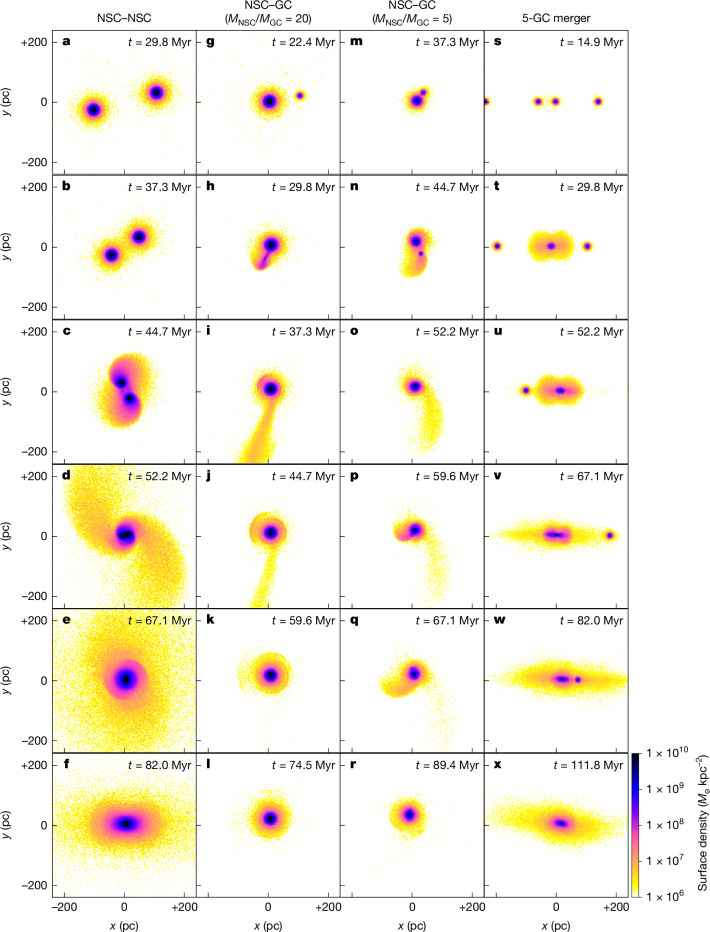
Snapshots illustrating the key steps of the N-body simulations of merging compact stellar objects. **a**–**f**, An NSC–NSC merger with non-null tangential velocity. **g**–**l**, An NSC–GC merger with a mass ratio 1:20. **m**–**r**, A 1:5 NSC–GC merger. **s**–**x**, a 5-GC merger. Assuming a stellar mass-to-light ratio of 7, a surface density of 3.1(1.2) × 10^6^ *M*_⊙_ kpc^−^^2^ translates in a surface brightness of 27(28) mag arcsec^−2^ (*r*-band filter).

To investigate the origin of the nuclear stellar tails, we compared our observations with the results of collisionless *N*-body simulations of NSC–NSC, NSC–GC and GC–GC mergers. We explored the effect of the simulation parameters—for example, the impact parameters, radial velocity, tangential velocity or mass ratio—on the merging process. Based on the average properties of NSCs in the MATLAS dwarfs observed with HST (M. Poulain et al., manuscript in preparation) and the properties of the Milky Way and the Andromeda galaxy GCs^22,23^, we used an initial stellar mass M_*_ = 10^6.5^*M*_⊙_ and 10^5.2^*M*_⊙_ for NSCs and GCs, respectively. Further details on the simulations can be found in the Methods. In Fig. 2, we show the main observed effects of these parameters on the different types of simulation. Our results indicate that overall, the merger of two star clusters can occur on short timescales, typically within a maximum of 50 Myr from first contact between the clusters to complete coalescence. We note the clusters must collide for the merger to happen on these short timescales. If the tangential or radial velocity is high enough so that they do not make contact, then they would orbit past each other without creating any features, and perhaps never merge.

The merger of clusters of similar mass (1:1), that is, two NSCs or two GCs, induces the formation of an extended elliptical nucleus without a long and extended tail, as represented in Fig. 2a–f. When the merger involves more than two GCs, as shown in snapshots Fig. 2s–x, the larger the number of clusters, the longer the extension of the final remnant nucleus. Moreover, an S-shape structure can be visible for a few tens of Myr when a non-null tangential velocity is applied to the colliding cluster, as observed in Fig. 2c,d.

An NSC–GC merger, or a merger of star clusters with mass ratios of at least 1:5 or greater, produces extended stellar tails together with shell structures around the newly formed NSC. A 1:20 and 1:5 merger are represented in snapshots Fig. 2g–l and Fig. 2m–r, respectively. Assuming an old red stellar population in the clusters and a detection limit of 28 mag arcsec^−2^, tails are visible for about 30–40 Myr. Shells at that surface brightness or brighter remain for a longer time (45–90 Myr). However, the detailed structures of the shells are more likely to appear diffuse at the resolution of HST, making them more difficult to identify in our observations.

The length of the tidal tail depends on the difference of mass between the two clusters, in which the larger the mass ratio, the longer the tail. For example, the maximum length of the tail changes from about 480 pc for a 1:20 mass ratio to only 275 pc for a 1:5 mass ratio. Moreover, in the case of small mass ratios (*M*_NSC_/*M*_GC_ = 5), we observe the apparition of a smaller secondary tail, as seen in Fig. 2p,q.

We note that a tangential velocity of 2 km s^−1^, or less, did not affect the overall morphology of the NSC–GC mergers. However, for a 1:20 ratio and a tangential velocity similar to the galaxy circular velocity (here 5 km s^−1^), multiple pericenter passages are necessary before the merger occurs, each of them producing a very short lasting (about 7.5 Myr) and small tail (around 100 pc at 28 mag arcsec^−2^), whereas the merger produces a longer tail (up to 250 pc) visible for about 38 Myr at 28 mag arcsec^−2^.

We further investigated the effect of the stellar and dark-matter components of the host galaxy on the formation of the tidal tails. The use of a cuspy halo or more massive disk slightly shortens the timescale on which the merger happens compared with our fiducial dwarf galaxy model, because of increased dynamical friction. But it has only a mild impact on the duration for which the streams will be visible. We observe that the tidal tails last up to 60 Myr at 28 mag arcsec^−2^ when using a 10 times more massive stellar disk. This suggests that the detection rate should increase with the stellar mass of the galaxy. However, we find that the tidal features, with their low surface brightness, will be more easily hidden by the intrinsic brightness of the higher surface brightness dwarfs, and thus be more difficult to detect in a typical dwarf than in UDGs. Overall, a change in the stellar or dark-matter components does not seem to have a huge impact on the general shape of the created tidal tails, and although the features will be longer detectable with the former, the timescales are still short, less than 100 Myr.

**Fig. 3:**
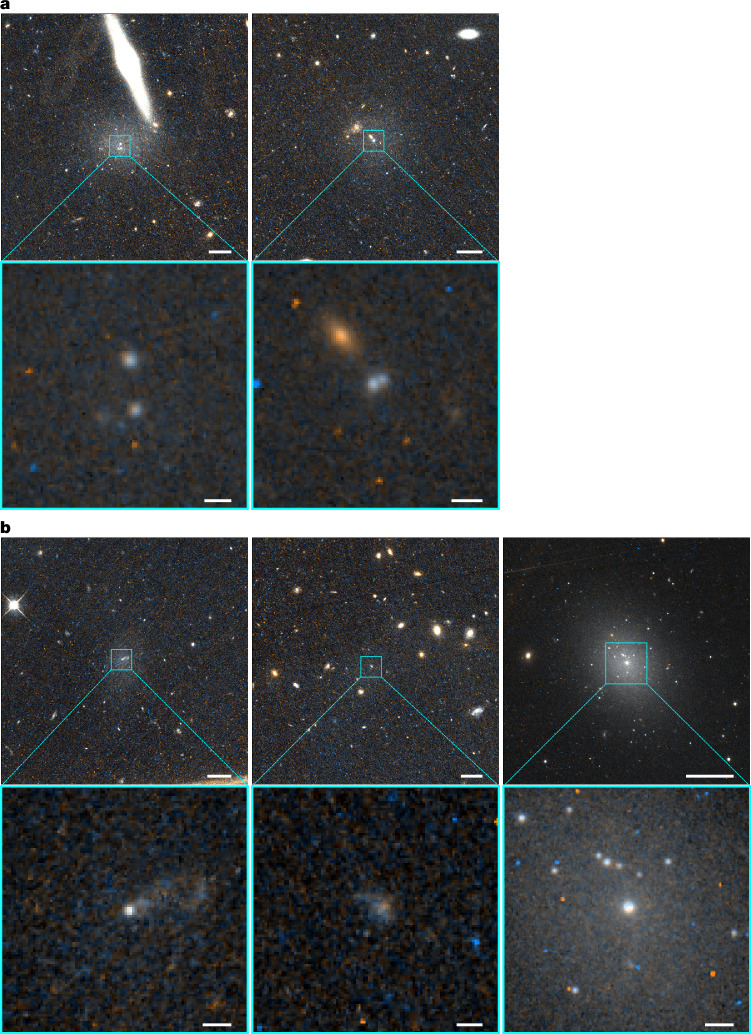
Main steps of the migration and merging of GCs in dwarf galaxies. **a**,**b**, The sequence starts with the migration of massive GCs towards the galaxy centre (**a**), as seen in the two double-nucleated dwarfs MATLAS-138 and MATLAS-987, followed by a GC–NSC merger that exhibits tidal tails (**b**), as in MATLAS-207, MATLAS-1216, and MATLAS-1938. We show 1′ × 1′ and 5″ × 5″ HST F606W and F814W colour-composed cutouts of the galaxies and their nuclear region, respectively. The magnification on MATLAS-1938 nucleus is of 10″ × 10″ to highlight the elongation of the stellar tail. Scale bars, 1 kpc (**a**, top; **b**, top); 100 pc (**a**, bottom; **b**, bottom).

Comparing the results of the photometric study of the central parts of the dwarf galaxies with the simulations allows us to reconstruct the two main steps of the migration scenario: the infall and merger of star clusters (Fig. 3). We observe a possible migration of massive GCs towards the galaxy centre of MATLAS-138 and MATLAS-987. Furthermore, given the bright nuclei and the large extent of the tails in MATLAS-207, MATLAS-1216 and MATLAS-1938, we probably witness the merging of two star clusters with a notable mass difference, such as NSC–GC mergers. Moreover, observations and simulations concur to highlight the rarity of detection of these complex nuclei, as suggested by the computed low detection rate and the short timescales of this formation channel. We also expect to see fewer tidal tails than star clusters in the nuclear region given their low surface brightness and the fact that they are only produced when the merger occurs between the star clusters. Therefore, large surveys of low surface brightness dwarf galaxies with deep and high-resolution images are required to observe these processes. We expect to find similar structures in upcoming large space telescope surveys, such as the Euclid Wide Survey^24^.

## Methods

### MATLAS

MATLAS^25,26^ is a deep optical imaging survey exploring the mass assembly of 180 massive early-type galaxies and the build-up of their scaling relations by studying their outermost stellar populations, their fine structures (for example, tidal tails, shells and stellar streams), their GC population and their dwarf galaxy satellites. The galaxies are located beyond the Local Volume, at distances between 10 Mpc and 45 Mpc and outside of galaxy cluster environments. The observations were taken with the MegaCam camera of the Canada France Hawaii Telescope between 2012 and 2015 and are composed of 12, 150, 148 and 79 images with a 1 deg^2^ field of view in the *u*-, *g*-, *r*-, *i*-band, respectively. The data were reduced with the help of the Elixir-LSB pipeline, providing a surface brightness as deep as 28.5–29 mag arcsec^−2^ in the *g*-band.

The MATLAS dwarf catalogue^27,28^ contains 2,210 galaxies identified using a visual inspection of the 150 fields in the *g*-band combined with a semi-automated catalogue generated using SOURCE EXTRACTOR^29^ parameters that was visually cleaned to reject potential background sources and to assign a morphological type to each dwarf candidate. The final classification resulted in a sample of 1,634 dwarf ellipticals (73%) and 576 dwarf irregulars (27%). During the morphological classification, we defined a nucleus as a bright, compact source located within 0.5*R*_e_ of the galaxy centre and brighter than any other compact sources within 1*R*_e_. This led to a sample of 507 nucleated galaxies. Unless a distance measurement was available, we assumed each dwarf is located at the distance of the central early-type galaxy targeted in the field in which the dwarf was detected.

### HST follow-up observations

Although the deep MATLAS ground-based images are ideal for detection of substructure with low surface brightness, the average image seeing (0.96″ in the *g*-band) limits our ability to resolve the GCs, NSCs and any related structures. Thus, high-resolution imaging from space-based observatories, such as the HST, is required. In that context, we conducted follow-up observations in the F606W and F814W filters of the HST ACS camera. The project was designed to characterize the GC population of UDGs^20^. Combining the Cycle 27 program GO-16082 (principal investigator O.M.) with the Cycle 28 and 29 snapshot programs SNAP-16257 and SNAP-16711 (principal investigator F.R.M.), we obtained ACS/WFC images for a sample of 79 dwarf galaxies. Given the science goals of the project, most of the sample has a low surface brightness and a large size typical of UDGs. Among the targets, we found 41 nucleated dwarfs, 13 of which are newly identified in the HST imaging. Serendipitously, we noticed that 10 nucleated dwarfs show a complex nuclear region composed of multiple star clusters and tidal tails.

### Dwarf–dwarf merger candidates

To ensure that our sample is composed of the most likely star cluster merger systems caused by the effect of dynamical friction on GCs alone, excluding any environmental influence on the galaxies, we searched for signs of past galaxy interactions in the light profile of the dwarfs, in particular in their surroundings. Observational signatures of these events include extended tidal tails, stellar shells or disturbances in the main body of the dwarf. Focusing on the sample with complex nuclei, six of the dwarfs exhibit no visible signs of interactions with nearby galaxies (Extended Data Fig. 1), implying that the nuclear structures most likely formed from internal processes, whereas four galaxies with asymmetric shapes appear to be tidally perturbed (Extended Data Fig. 2). Some of the latter disturbances could be because of a tidal interaction with their massive host galaxy. Studies^30,31^ reporting tidal interactions between dwarfs and massive galaxies typically find spatial separations between the galaxies within 100 kpc and the presence of a tidal bridge. For our disturbed objects, the projected distance to the host lies beyond 100 kpc. Moreover, none of them exhibit tails pointing towards the host. We also investigated the possibility that they could be satellites of another massive galaxy. However, the smallest physical separations—calculated assuming the dwarf is at the same distance as the potential massive host—are all greater than 100 kpc. Therefore, we posit that these disturbances are the result of a dwarf–dwarf interaction or merger. Based on this, we excluded these objects from our sample, leaving us with the six candidates whose complex nuclei probably formed from an internal process.

### Complex nuclei photometry

**Extended Data Fig. 3:**
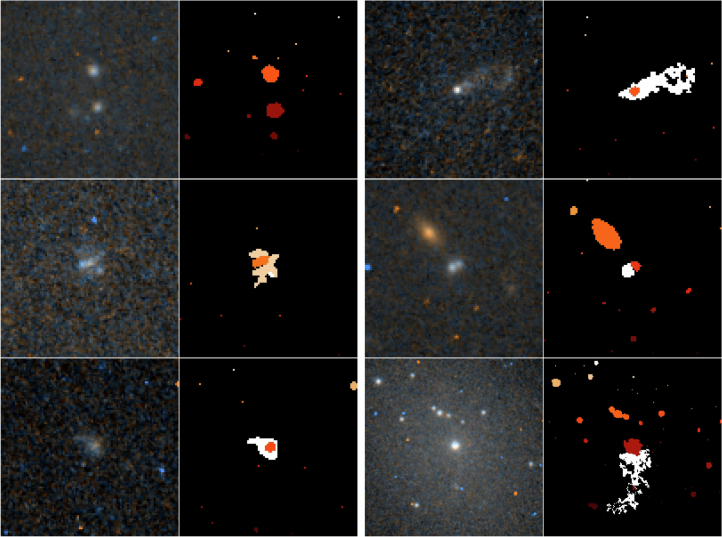
Composed segmentation images from MTO displaying the detection of the different structures within the nuclear region of the dwarfs. *From top to bottom*, *left to right*: MATLAS-138, MATLAS-207, MATLAS-658, MATLAS-987, MATLAS-1216, MATLAS-1938.

We carried out a photometric study of the nuclear substructures in the six non-interacting dwarfs identified in the HST images. The extracted properties of these objects were then compared with those of GCs and NSCs identified in other MATLAS dwarfs^20^ and for which photometry could be reliably extracted. To disentangle the different substructures of the complex nuclear regions and measure their photometry, we made use of the software MTObjects^32^ (MTO). MTO is a Max-Tree-based method optimized to detect sources with low surface brightness. We ran the software on each of the complex nuclear regions to produce a segmentation image of the structures. Image segmentation deblends the different sources in an image by grouping the pixels belonging to each object under the same flag. We produced segmentation images on both the F606W and F814W images to ensure all the structures were successfully detected. For all nuclear regions, we merged the obtained segmentation maps to generate a final image, including the regions of all the structures (Extended Data Fig. 3). Using the final segmentation images, we then derived the magnitude and colour of each nuclear substructure from the F606W and F814W images. We derived errors on the magnitude by combining the standard error with the standard deviation of the sky. The derived properties are available in Extended Data Table 1. All the nuclear structures have colours in the range of the GCs in the MATLAS dwarfs observed with HST^20^, except for one substructure. The substructure is found in a dwarf that exhibits a star cluster with a red colour (*m*_F606W_ − *m*_F814W_)_0_ = 1.23 ± 0.05 in the typical range of GCs, together with a faint blue stellar tail and clump with 0.03 ± 0.05 and  −0.43 ± 0.31, respectively, which is similar to some star-forming nuclei in dwarf elliptical galaxies^33^ that can be related to a wet-merger scenario. Although an Hi study of the MATLAS dwarfs^34^ reported no Hi detection in this galaxy, we opted to remove it from the sample.

### *N*-body simulations

We performed collisionless *N*-body simulations of star cluster mergers at the centre of a dwarf galaxy. Our method has similarities with previous simulations of star cluster mergers in spiral galaxies^35^, which we adapted to our HST sample of dwarf galaxies. The setup of these simulations is designed to model the final stages of the merger. Thus, in their current form, these simulations cannot be used to model the full orbital decay of the star clusters.

We chose to model a dwarf with a cored dark-matter profile using a spherical Burkert halo of mass *M*_h_ = 3 × 10^10^*M*_⊙_ and a scale radius *R*_0_ = 5.6 kpc such that the halo radius is *R*_h_ = 3.4*R*_0_ (ref. ^36^). The dwarf also has a double exponential stellar disk of mass log(*M*_⋆_/*M*_⊙_) = 7.7, *R*_e_ = 1.5 kpc and an axial ratio of 0.6 (that is, a thick disk), corresponding to the median *M*_⋆_ and *R*_e_ of the MATLAS dwarfs observed by HST. In this way, the merging clusters also interact gravitationally with both the dark-matter halo and stellar disk of the model dwarf galaxy. The halo consists of 10^7^ dark-matter particles of mass 300*M*_⊙_, as well as just more than 10^5^ stellar particles of mass 500*M*_⊙_. Furthermore, we tested the effect of a change in the stellar component with a 10 times more massive stellar disk. We also modified the dark-matter profile by switching from a cored Burkert to a cuspy NFW halo of equal halo mass and NFW concentration *c* = 11, typical for this mass^37^.

We simulated three types of cluster mergers: NSC–NSC, NSC–GC and GC–GC. We use a Sérsic model for the NSC and GC. We set the Sérsic parameters of the NSC profile according to the average properties of our HST sample, such that log(*M*_⋆_/*M*_⊙_) = 6.5, *R*_e_ = 7 pc and Sérsic index *n* = 2. The Sérsic parameters of the GC are based on the average parameters of the GCs in Andromeda and the Milky Way. That is log(*M*_⋆_/*M*_⊙_) = 5.2, *R*_e_ = 3.2 pc and *n* = 1. These Sérsic models consist of 5 × 10^5^ stars of mass 6.3*M*_⊙_ and 10^5^ stars of mass 1.6*M*_⊙_ for the NSC and GC, respectively. In the case of an NSC–NSC merger or GC–GC merger, the merger mass ratio is 1:1. We also test the case of multiple sequential GC mergers until up to five GCs have been merged into a single remnant. For NSC–GC mergers, we test mass ratios of 5, 10 and 20.

The simulations were conducted using the adaptive mesh refinement code RAMSES^38^. The simulation volume has a side length of 200 kpc, which encompasses the entire dark-matter halo of the model galaxy. We choose a refinement grid in which the maximum level of refinement varies with distance from the dwarf galaxy centre. Inside of 0.8 kpc, which corresponds to the region in which the clusters merge and interact with the dwarf galaxy mass distribution (the merger region), a resolution of 0.375 pc is reached. Outside of the merger region, the resolution steadily steps down (at radii of 2, 10, 20, 40, 80 and 160 kpc) reaching a minimum resolution of 6.25 kpc in the far outskirts of the dark-matter halo. In this way, we can model the dynamical evolution of the entire galaxy, while restricting the region of maximum resolution to the area in which it is required. We also conducted several simulations at a lower resolution of 1.5 pc. We find this resolution change does not affect our main conclusions.

The simulations are performed over time durations ranging from 75 Myr to 278 Myr, depending on the time required for the merger process to be fully completed. Snapshots were produced frequently, with an output every 3.73 Myr to capture the rapidly changing details of the merger process. Typically, a star cluster (either NSC or GC) collides with another cluster located at the centre of the dwarf. We set the initial position of the infalling star cluster to be 200 pc from the galaxy centre. A fiducial radial velocity of 2.5 km s^−1^ and a tangential velocity of 1.5 km s^−1^ is chosen. However, we also tested the impact of increasing the radial velocity up to 5, 10, 25 and 50 km s^−1^ and varying the tangential velocity from 0 to 0.5, 1.5, 2, 5 and 7.5 km s^−1^, to look for possible impacts on the structures formed during the merger process. The tangential velocity of 5 km s^−1^ is similar to the circular velocity of the dwarf galaxy model at the radius for which the star clusters are interacting. Overall, as long as the two clusters end up colliding, we find that the appearance and duration of these structures are not strongly affected by these choices. The two exceptions are the highest radial and tangential velocities considered, for which no merger happens within 0.5 Gyr and flybys occur without creating any tidal features.

## Online content

Any methods, additional references, Nature Portfolio reporting summaries, source data, extended data, supplementary information, acknowledgements, peer review information; details of author contributions and competing interests; and statements of data and code availability are available at 10.1038/s41586-025-08783-9.

## Figures and Tables

**Extended Data Table 1:**
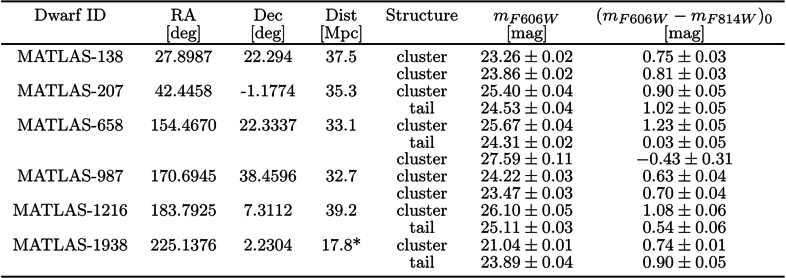
Derived photometric properties

* Distance measurement from SDSS DR13 database.

The magnitudes and colors are extinction corrected, using the NASA/IPAC Extragalactic Database (NED) Galactic Extinction Calculator.

## Data Availability

The HST data used in this analysis are available at the Mikulski Archive for Space Telescopes with the programme IDs GO-16082, SNAP-16257 and SNAP-16711.

## Code availability

This work made use of the software MTObjects and widely used packages of Python 3 such as Matplotlib, NumPy, SciPy and Astropy. The simulations are based on DICE for galaxy model setup, RAMSES to run it and RDRAMSES for analysis. All codes and software are publicly available.
